# A Rare but Critical Presentation in Primary Care: A Case Report on Unilateral Ureterolithiasis Leading to Postrenal Acute Kidney Injury

**DOI:** 10.7759/cureus.104362

**Published:** 2026-02-27

**Authors:** Melis Dorter, Ahmad Eid, Naela Darwish Saad

**Affiliations:** 1 Emergency Medicine, Primary Health Care Corporation, Doha, QAT; 2 Family Medicine, Primary Health Care Corporation, Doha, QAT

**Keywords:** acute kidney injury, emergency medicine, obstructive uropathy, primary care, s: urolithiasis, ureteral stone

## Abstract

Distal ureteral calculi measuring ≤5 mm are generally expected to pass spontaneously and are commonly managed conservatively. However, persistent obstruction may occur and can result in renal impairment. A 31-year-old previously healthy male presented with lower abdominal discomfort, dysuria, and penile pain. Initial noncontrast computed tomography (CT) revealed a 4 mm calculus at the left ureterovesical junction without hydronephrosis. He was discharged with conservative management, including nonsteroidal anti-inflammatory drugs (NSAIDs), oral cefixime 400 mg daily, hydration advice, and follow-up instructions. Ten days later, he re-presented with severe flank pain and reduced urine output. Laboratory evaluation demonstrated elevated serum creatinine consistent with acute kidney injury (AKI). Repeat CT showed the persistent distal ureteral stone with new hydronephrosis and periureteric fat stranding. The patient underwent urgent placement of a double-J ureteral stent, resulting in rapid clinical improvement and normalization of renal function within four days. Although small distal ureteral stones are often considered low risk, this case demonstrates that even a 4 mm calculus may lead to persistent obstruction and postrenal AKI. Clinicians should remain cautious for complications in patients with ongoing symptoms and consider timely reassessment and repeat imaging when clinical deterioration occurs.

## Introduction

Urolithiasis is a common condition worldwide, with a lifetime prevalence estimated at approximately 12% in men and 6% in women [1,2]. Recurrence rates approach 50% within 10 years [1]. Established risk factors include male sex, increasing age, obesity, metabolic syndrome, dehydration, and dietary factors [1,3].

Small ureteral calculi, defined as stones measuring ≤5 mm in maximal diameter, are generally expected to pass spontaneously without intervention [2,3]. Reported spontaneous passage rates for distal ureteral stones of this size range from 90% to 98% [3-5]. Consequently, conservative management with analgesia and observation is recommended in clinically stable patients without infection, refractory pain, or renal impairment [4,5].

Despite these expectations, stone size alone does not reliably predict outcome. Factors such as impaction, duration of obstruction, and anatomical location may contribute to persistent obstruction and renal dysfunction [3,5]. Postrenal acute kidney injury (AKI) is most commonly associated with bilateral obstruction or obstruction in a solitary functioning kidney [6]. However, case reports have described complications arising from small distal ureteral stones, including spontaneous forniceal rupture and ureteral perforation [7-9]. Significant renal impairment secondary to unilateral distal obstruction is less frequently reported, and when present, typically involves delayed recognition or prolonged obstruction. This case describes delayed postrenal AKI caused by a small distal ureteral stone despite initially reassuring imaging findings, underscoring the importance of vigilant follow-up and reassessment when symptoms persist.

## Case presentation

A 31-year-old South Asian male with no significant past medical history presented to a primary care clinic with a three-day history of lower abdominal discomfort, dysuria, and prominent penile pain. He denied fever, nausea, vomiting, gross hematuria, or urinary retention. There was no known history of nephrolithiasis, chronic kidney disease, diabetes, hypertension, or recent dehydration.

Vital signs were stable: blood pressure, 116/75 mmHg; heart rate, 72 beats per minute; respiratory rate, 17 breaths per minute; temperature, 36.7°C; and oxygen saturation, 100% on room air. Clinical examination revealed mild left flank tenderness without abdominal guarding.

Laboratory evaluation demonstrated a white blood cell count of 12.2 × 10⁹/L and serum creatinine of 82 µmol/L (estimated glomerular filtration rate (eGFR) >90 mL/min/1.73 m²), confirming normal baseline renal function. Urinalysis showed microscopic hematuria without nitrites or leukocyte esterase. There was no laboratory evidence of urinary tract infection (UTI).

**Figure 1:**
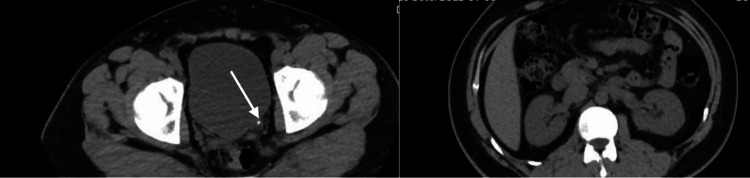
Noncontrast computed tomography of the urinary tract demonstrating a 4 mm calculus at the left ureterovesical junction (arrow), without hydronephrosis

Noncontrast computed tomography (CT) of the urinary tract revealed a 4 mm calculus at the left ureterovesical junction (UVJ) without hydronephrosis and with normal contralateral renal appearance (Figure 1). No cortical thinning or delayed excretion was noted.

Given the small stone size (≤5 mm) and the absence of hydronephrosis or renal impairment, conservative management was initiated. The patient was discharged with oral nonsteroidal anti-inflammatory drugs (NSAIDs) for analgesia, oral cefixime 400 mg daily, hydration advice, and instructions for reassessment if symptoms worsened or urine output decreased.

Ten days later, the patient re-presented with severe left flank pain and reduced urine output, which he described as decreased frequency and volume compared to baseline. He remained hemodynamically stable without signs of hypovolemia.

Repeat laboratory testing demonstrated leukocytosis (13.2 × 10⁹/L) and an elevated serum creatinine of 144 µmol/L (eGFR: 55 mL/min/1.73 m²), meeting criteria for AKI. Electrolytes were within normal limits, and he did not require hemodialysis. No alternative nephrotoxic exposures beyond prescribed NSAIDs were identified.

**Figure 2:**
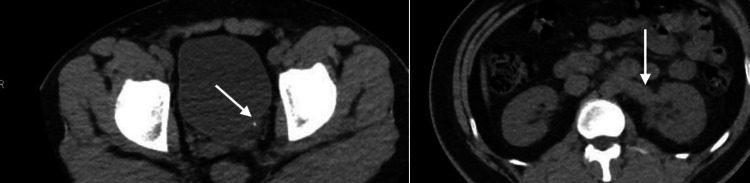
Repeat noncontrast computed tomography performed 10 days later showing the same 4 mm calculus at the left ureterovesical junction (arrow), now with left-sided hydronephrosis and periureteric fat stranding

Given persistent symptoms and renal deterioration, he was urgently referred to the emergency department and admitted under the urology service. Repeat CT demonstrated the persistent 4 mm distal ureteral stone with new mild-to-moderate left hydronephrosis and periureteric fat stranding (Figure 2). The contralateral kidney remained normal.

The patient underwent cystoscopic placement of a double-J ureteral stent under general anesthesia for urinary decompression. Following the intervention, urine output improved, and flank pain resolved. Serum creatinine progressively normalized, returning to baseline (85 µmol/L) by postoperative day four.

He was discharged hemodynamically stable, pain-free, and with normalized renal function. Follow-up at four weeks confirmed preserved renal function and planned definitive stone management. The clinical timeline and laboratory progression are summarized in Tables 1-2. 

**Table 1:** Laboratory investigations at the initial and repeat presentations eGFR: estimated glomerular filtration rate

Parameter	Reference range	Initial presentation	10-day re-presentation
White blood cell count (×10⁹/L)	4.0-10.0	12.2	13.2
Hemoglobin (g/dL)	13-17	14.1	Not repeated
Serum creatinine (µmol/L)	60-110	82	144
eGFR (mL/min/1.73 m²)	>90	>90	55
Urinalysis	-	Microscopic hematuria	Not documented

**Table 2:** Clinical timeline and management course UVJ: ureterovesical junction; ED: emergency department; NSAID: nonsteroidal anti-inflammatory drugs

Time point	Clinical status	Serum creatinine (µmol/L)	Imaging findings	Management
Day 0	Initial presentation	82	4 mm left UVJ stone, no hydronephrosis	NSAIDs, oral cefixime, hydration
Day 10	Re-presentation with reduced urine output	144	Persistent stone	Urgent referral to ED
Day 10	Hospital admission	144	Mild–moderate hydronephrosis	Double-J stent placement
Day 14	Poststent recovery	85	Not repeated	Clinical improvement
4-week follow-up	Outpatient review	Normal	Not indicated	Planned definitive management

## Discussion

Distal ureteral stones measuring ≤5 mm are widely regarded as low-risk lesions due to high spontaneous passage rates [3-5]. Conservative management is therefore considered appropriate in carefully selected patients [4,5]. However, persistent obstruction may occur despite a small stone size. The pathophysiology of obstructive nephropathy involves elevated intratubular pressure, decreased renal perfusion, and inflammatory injury, which may lead to progressive renal dysfunction if obstruction is not relieved [6]. Although postrenal AKI most commonly results from bilateral obstruction or obstruction in a solitary kidney [6], unilateral obstruction can impair renal function when prolonged or associated with significant intrarenal pressure changes.

Previous reports have documented serious complications from small distal stones, including spontaneous forniceal rupture and ureteral perforation [7-9]. In these cases, stone size did not reliably predict clinical severity. While significant AKI secondary to unilateral distal obstruction remains less commonly described, delayed recognition of persistent obstruction has been implicated in renal impairment.

In the present case, alternative causes of AKI were considered. Baseline renal function was normal. There was no evidence of systemic infection, hypotension, or hypovolemia. Electrolytes were stable, and dialysis was not required. The contralateral kidney was radiographically normal. Although NSAIDs were prescribed, the temporal association of worsening obstruction on repeat imaging with concurrent hydronephrosis supports obstructive pathophysiology as the primary mechanism.

Current urological guidelines recommend reassessment and consideration of repeat imaging in patients with persistent pain or worsening renal function despite conservative management [4,5]. In this case, repeat imaging was pivotal in identifying new hydronephrosis and confirming persistent obstruction, prompting timely intervention. From a clinical standpoint, this case highlights that stone size alone should not determine reassurance. Persistent symptoms, reduced urine output, or biochemical deterioration warrant prompt reassessment and possible escalation of care.

## Conclusions

This case illustrates that small distal ureteral stones may still result in clinically significant obstruction and postrenal AKI despite initially reassuring imaging findings. Persistent symptoms or reduced urine output following conservative management should prompt reassessment and repeat imaging. Stone size alone should not determine clinical reassurance, and appropriate safety-netting and follow-up are essential to prevent delayed recognition of obstruction and renal impairment.
